# Effect of high‐intensity interval training on hippocampal metabolism in older adolescents

**DOI:** 10.1111/psyp.14090

**Published:** 2022-05-22

**Authors:** Sarah Ruth Valkenborghs, Charles H. Hillman, Oun Al‐Iedani, Michael Nilsson, Jordan J. Smith, Angus Aaron Leahy, Simon K. Harries, Saadallah Ramadan, David Revalds Lubans

**Affiliations:** ^1^ School of Biomedical Sciences and Pharmacy The University of Newcastle Callaghan New South Wales Australia; ^2^ Centre for Active Living and Learning The University of Newcastle Callaghan New South Wales Australia; ^3^ Center for Cognitive & Brain Health, Department of Psychology, Department of Physical Therapy, Movement, and Rehabilitation Sciences Northeastern University Boston Massachusetts USA; ^4^ School of Health Sciences The University of Newcastle Callaghan New South Wales Australia; ^5^ Centre for Rehab Innovations The University of Newcastle Callaghan New South Wales Australia; ^6^ Priority Research Centre for Stroke and Brain Injury The University of Newcastle Callaghan New South Wales Australia; ^7^ School of Medicine and Public Health The University of Newcastle Callaghan New South Wales Australia; ^8^ School of Education The University of Newcastle Callaghan New South Wales Australia

**Keywords:** adolescents, cognition, exercise, working memory

## Abstract

Although well‐evidenced in older adults, the effects of exercise on the hippocampus in youth are relatively unknown. This study examined the impact of a 6‐month school‐based physical activity intervention on hip**p**ocampal metabolism in adolescents using magnetic resonance spectroscopy. A subset of lower fit older adolescents [*N* = 56, 61% female, 16.1 ± 0.4 years] was included from four secondary schools (10 classes) in New South Wales, Australia, who were participating in a larger cluster randomized controlled trial. Participants were randomized to the Burn 2 Learn (B2L) intervention (five classes, 30 participants) or a control group (five classes, 26 participants). Changes in hippocampal metabolism were assessed using linear mixed models adjusted for clustering at the class level. We observed group‐by‐time effects for the B2L intervention on *N*‐acetylaspartate (NAA) (+2.66 mmol/L, 95% CI 0.20 to 5.11, *d* = 0.66) and glutamate+glutamine (Glx) (+3.38 mmol/L, 95% CI 0.34 to 6.42, *d* = 0.67) in the left hippocampus. Increases in left hippocampal NAA and Glx concentrations were associated with improvements in cardiorespiratory fitness (NAA: *r*_*s*_ = 0.52, *p* = .016; Glx: *r*_*s*_ = 0.57, *p* = .007), lower body muscular fitness (NAA: *r*_*s*_ = 0.49, *p* = .018; Glx: *r*_*s*_ = 0.59, *p* = .003), and working memory (NAA: *r*_*s*_ = 0.42, *p* = .032; Glx: *r*_*s*_ = 0.43, *p* = .028) in the intervention group. Our findings suggest physical activity may improve hippocampal metabolism in lower fit older adolescents with implications for working memory. Further studies involving larger samples are needed to replicate our findings.

## Abstract 2

Participation in physical activity has small positive effects on measures of brain and cognition in youth, but the mechanisms are not well understood. This is the first study to examine the effects of an exercise intervention on adolescents' hippocampal metabolism using magnetic resonance spectroscopy and provides evidence for potential mechanisms linking participation in physical activity with enhanced executive function.

**Abbreviations**
3D: three dimensional
AP: anterior–posterior
B2L: Burn 2 Learn
BMI: body mass index
CC: cranio‐caudal
CRF: cardiorespiratory fitness
CVD: cardiovascular disease
CSF: cerebrospinal fluid
FOV: field of view
FWHM: full‐width at half‐maximum
Glx: glutamate+glutamine
HF: head‐feet
HIIT: high‐intensity interval training
HR_max_
: heart rate maximum
Hz: Hertz
LASER: localized by adiabatic selective refocusing
LCModel: linear combination of model spectra
MCI: mild cognitive impairment
mmol: millimole
MPRAGE: magnetization‐prepared rapid acquisition with gradient echo
MRI: magnetic resonance imaging
MRS: magnetic resonance spectroscopy
MVPA: moderate‐vigorous physical activity
Myo: Myo‐inositol
NAA: *N*‐acetylaspartate
NSW: New South Wales
RCT: randomized controlled trial
RL: right‐to‐left
SNR: signal‐to‐noise ratio
tCho: total choline
tCr: total creatine
TE: echo time
TI: inversion time
TR: repetition time
TV: transverse
VOI: volume of interest

## 1 INTRODUCTION

Participation in physical activity of sufficient volume and intensity to improve and maintain health‐related fitness is essential for current and future health (Lang et al., 2018). Cardiorespiratory fitness (CRF) is inversely associated with cardio‐metabolic risk factors during adolescence and future burden of disability (Henriksson et al., 2021; Raghuveer et al., 2020). Of concern, most school‐aged children and adolescents are not sufficiently active and there has been a secular decline in CRF (Tomkinson et al., 2019).

Accumulating evidence supports the benefits of physical activity and fitness for young people's cognitive function (Alvarez‐Bueno et al., 2017; Leahy et al., 2020; Sun et al., 2021). Despite exponential growth of research in this area, the neurobiological mechanisms mediating the cognitive benefits of physical activity are not well established (Lubans et al., 2016; Valkenborghs et al., 2019). Stemming from fundamental animal models, the hippocampus is the most consistently studied and well‐evidenced human brain region in physical activity research. Of note, cross‐sectional studies have demonstrated that hippocampal volume mediates the association between CRF and memory in children and adults (Chaddock et al., 2010; Erickson et al., 2009). More recently, several meta‐analyses have confirmed the plasticity of the hippocampus in response to physical activity interventions in adults. Specifically, exercise appears to attenuate the age‐related volumetric loss known to precede and lead to cognitive decline and dementia (Firth et al., 2018; Jack et al., 2010; Ji et al., 2021; Wilckens et al., 2021).

Surprisingly, our recent systematic review of magnetic resonance imaging (MRI) studies failed to identify any experimental studies reporting the effects of physical activity on hippocampal volume in youth (Valkenborghs et al., 2019). However, increases in hippocampal volume have been observed in young adults (Thomas et al., 2016), confirming that the hippocampus is responsive to physical activity in younger people. Physical endurance and skill training also increase hippocampal neurogenesis in an animal model of adolescence (DiFeo & Shors, 2017). Taken together, it is highly plausible that physical activity may confer beneficial effects on the human hippocampus during adolescence. While it remains to be established if physical activity can increase the volumetric growth of the hippocampus during childhood and adolescence in humans, several studies have reported associations between CRF and hippocampal volume (Chaddock et al., 2010; Esteban‐Cornejo et al., 2017; Herting & Nagel, 2012). Therefore, it may be that changes in hippocampal volume are only elicited by physical activity of sufficient volume and intensity to improve CRF, as seems to be the case for other structural brain changes (e.g., white matter integrity) (Valkenborghs et al., 2019).

While more‐easily investigated in animals, the experimental manipulation and measurement of microscopic neural mechanisms are difficult in in vivo human studies. However, magnetic resonance spectroscopy (MRS) may serve as a sensitive and stable measurement approach to non‐invasively monitor in vivo changes in markers of morphological adaptations (Brand et al., 1993; Guimaraes et al., 1995; Rae, 2014). *N*‐acetylaspartate (NAA) is considered a neuronal marker as it is synthesized in neurons and is mostly localized to cell bodies where it has a multi‐factorial role in lipid synthesis and myelination (Rae, 2014). Glutamate+glutamine (Glx), a class of excitatory amino acid, is considered a marker of brain metabolic activity as it plays a crucial role in mitochondrial metabolism and is involved in the maintenance and regulation of synaptic information transmission (Huang et al., 2017). Hippocampal concentrations of NAA and Glx typically decrease during aging (Huang et al., 2017; Rae, 2014; Yang et al., 2015), as well as in a range of pathologies including mild cognitive impairment (MCI), Alzheimer's disease, and mental health disorders (Deicken et al., 2003; Foy et al., 2011; Karl & Werner, 2010; Rauchmann et al., 2020; Rosso et al., 2017; Su et al., 2016). Similarly, hippocampal concentrations of total choline (tCho; a marker of phospholipid membrane levels/cell density) are decreased in Alzheimer's disease, mental health disorders, and former athletes with a history of concussion (Ende et al., 2000; Su et al., 2016; Tremblay et al., 2013), while higher levels have been observed in occipito‐parietal gray matter of endurance‐trained compared to sedentary adults (Gonzales et al., 2013). Poor body composition, cardiovascular health, and mental health are associated with decreased hippocampal concentrations of total creatine (tCr; a marker of metabolic activity) (Chiappelli et al., 2019; Coplan et al., 2014). In contrast, increased hippocampal concentrations of myo‐inositol (Myo; a marker of gliosis) have been observed with aging, and in former athletes with a history of concussion in which it was inversely correlated with memory (Brand et al., 1993; Tremblay et al., 2013; Yang et al., 2015).

Previous MRS studies have detected changes in hippocampal metabolism in response to physical activity interventions in young adult and clinical populations (Pajonk et al., 2010; Wagner et al., 2015). However, to our knowledge, no studies have used MRS to elucidate the effects of physical activity on hippocampal metabolism in a pediatric population (Valkenborghs et al., 2019). Therefore, the aim of this study was to examine the impact of a 6‐month physical activity intervention, involving high‐intensity interval training (HIIT), on hippocampal metabolism in lower fit older adolescents using MRS. We hypothesized that HIIT would increase concentrations of NAA, Glx, tCr, and tCho and decrease concentrations of Myo. While the hippocampus is not generally regarded as a primary node within the working memory network, it plays a role in many memory functions and is implicated in many disorders associated with memory impairments. Furthermore, hippocampal volume is associated with visual working memory (2‐back task) performance in young adults (Zhu et al., 2017). Pediatric studies have identified relationships between hippocampal functional connectivity and working memory performance (Liu et al., 2021) and fitness, relational memory, and hippocampal volume (Chaddock et al., 2010). Hence, as exploratory analyses, we hypothesized that improvements in measures of working memory performance, cardiorespiratory fitness, muscular fitness, physical activity, body composition, and sedentary time would be positively associated with increases in hippocampal concentrations of NAA, Glx, tCR, and tCho, and negatively associated with changes in hippocampal concentrations of Myo.

## 2 METHOD

### 2.1 Study design

This study involves a subset of participants from the Burn 2 Learn (B2L) cluster randomized controlled trial (RCT). Detailed descriptions of the study protocol and findings have been published previously (Leahy et al., 2019; Lubans et al., 2021). In the larger trial, we observed significant group‐by‐time effects for CRF and a range of secondary outcomes (e.g., muscular fitness and hair cortisol concentrations). Weight status moderated the effect of the intervention on working memory, with stronger effects observed among adolescents who were overweight or obese. This sub‐study involved older adolescents (*N* = 56) from four government secondary schools (10 classes) in New South Wales (NSW), Australia. School principals, teachers, parents, and students all provided informed written consent/assent prior to enrolment. Baseline data collection and teacher training occurred in the school term preceding intervention delivery (i.e., February–April 2018). Six‐month follow‐up data collection occurred from August–September 2018). The trial was prospectively registered with the Australian New Zealand Clinical Trials Registry (ACTRN12618000293268) and the design, conduct, and reporting adheres to the CONSORT and TIDier checklists (Campbell et al., 2012; Hoffmann et al., 2014). This study was approved by the University of Newcastle (H‐2016–0424) and the NSW Department of Education (SERAP: 2017116) human research ethics committees.

### 2.2 Sub‐study schools and participants recruitment

This sub‐study included a prospectively defined targeted population of participants recruited from 10 classes (5 × intervention, 5 × control) across four schools (2 × intervention, 2 × control) pair‐matched according to geographic location, school area‐level socioeconomic status, and student population educational advantage. These schools were identified pragmatically on the basis that they were within proximity to the Hunter Medical Research Institute to enable students to be scanned within time constraints of the school day. Individual participants (*N* = 56; *n* = 30 intervention; *n* = 26 control) were identified as being in the bottom 50% of students from their school for CRF (using their baseline multistage fitness test result), with the rationale that they would likely be most susceptible to increases in fitness and subsequently changes in hippocampal metabolism. These individuals were invited to undergo MRI assessments at baseline and 6‐months and were provided with a $50 gift card incentive at each visit.

### 2.3 Intervention

A detailed description of the B2L intervention has been published previously (Leahy et al., 2019). Briefly, teachers randomized to the intervention group received training, resources, and support to facilitate the delivery of HIIT during academic lessons. The intervention also included: (i) a seminar focused on the benefits of HIIT for students, (ii) purpose‐built smartphone application and heart rate monitors to support HIIT delivery, and (iii) e‐newsletters (i.e., information videos) for parents. Teachers were asked to facilitate at least two HIIT sessions per week for 16 weeks. The duration of HIIT sessions ranged from 8–20 min (including warm‐up and cool down), and involved a combination of aerobic (e.g., shuttle runs, jumping jacks) and body weight resistance exercises (e.g., push‐ups, squat jumps). Students were encouraged to reach ≥85% of their age‐predicted HR_max_ utilizing the B2L smartphone app and heart rate monitors.

### 2.4 Assessments

#### 2.4.1 Magnetic resonance spectroscopy

MRI scans were conducted on a 3.0 Tesla Prisma (Siemens Healthineers, Erlangen, Germany) scanner with a 64‐channel head and neck coil located at Hunter Medical Research Institute. Structural images were acquired using a 3D T1‐weighted magnetization‐prepared rapid gradient‐echo protocol with TR/TE/TI = 2000/2.96/900 ms, 9‐degree flip angle, FOV = 256 × 256 mm^2^, voxel size = 1 × 1 × 1 mm^3^, averages = 1 and acquisition time = 4 min 32 s.

**FIGURE 1:**
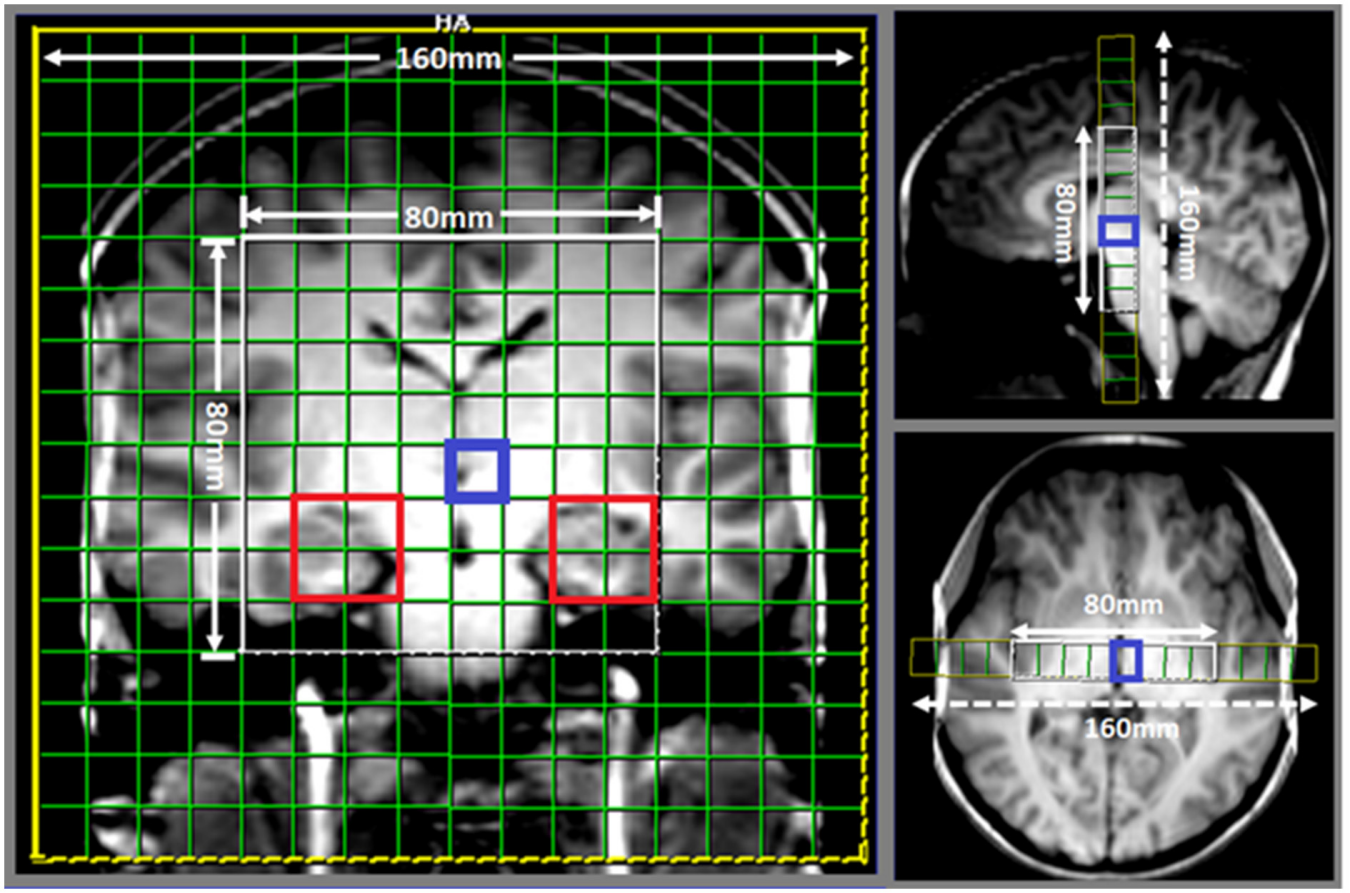
Position of the coronal 15 mm slice with voxel size of 15 × 10 × 10 mm^3^ (AP‐RL‐HF) within the VOI shown in axial, sagittal, and coronal T1 MPRAGE planes with the bilateral hippocampus highlighted in red

MRS imaging data were acquired using a product semi‐LASER sequence (Scheenen et al., 2008), with the following parameters: TR/TE: 1500/40 ms, averages = 3, vector size: 1024 points, voxel size: 15 × 10 × 10 mm^3^, delta frequency (offset): −2.7 ppm, water suppression enabled, VOI in (AP‐RL‐HF): 15 × 80 × 80 mm^3^, and FOV: 160 × 160 mm^2^ with slab thickness of 15 mm, phase‐encoding: weighted, matrix: 16 × 16, and acquisition time = 6 min 5 s. Unsuppressed water reference scans were acquired from the same FOV for metabolite quantification and eddy current corrections using above parameters, but with averages = 1 (duration 3 min 50 s). The VOI was placed from the mid corpus callosum to the pons on a sagittal T1 MPRAGE acquisition and included contributions from the frontal lobes, basal ganglia, medial temporal lobes, hippocampi, lateral ventricles, and pons. The VOI is bounded laterally by the medial temporal lobes and frontal white matter, superiorly by the mid‐corpus callosum, inferiorly by the pons, and is centered at the level of the third ventricle (15 × 80 × 80 mm, AP × TV × CC). A single 15 mm slice with voxel size of 15 × 10 × 10 mm^3^ within the VOI is shown in axial, sagittal, and coronal T1 MPRAGE planes in (Figure 1) with red boxes identifying the specific hippocampal voxels that data were extracted from and analyzed.

**FIGURE 2:**
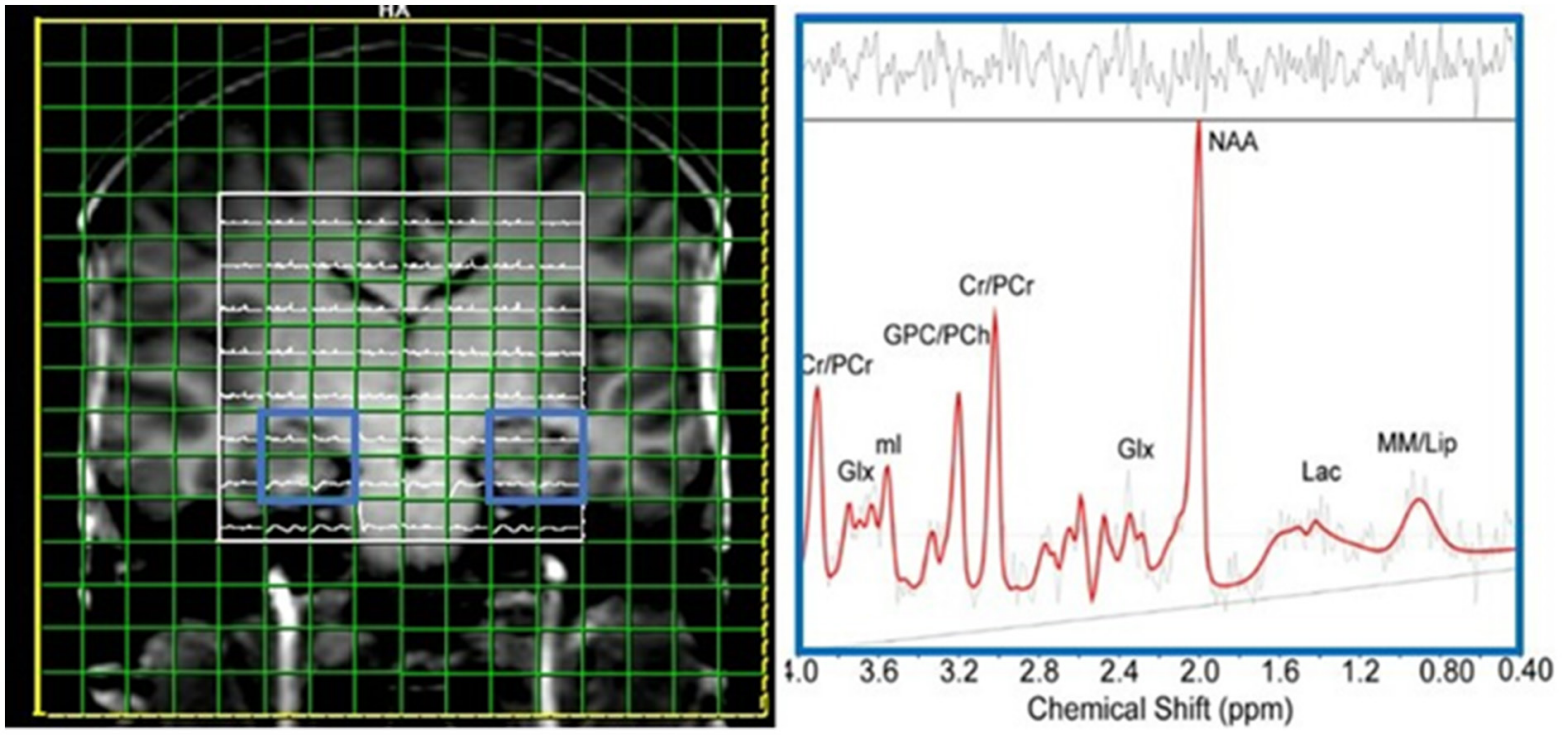
Spectra modeling in LCModel shows the fit‐curve calculated to estimate the chemical shift of signal peaks corresponding to molecular structure and the peak intensity corresponding to metabolite concentration

MRS data from the hippocampal voxels were analyzed using an automated linear combination of model spectra (LCModel) with a custom‐made basis set for the LASER sequence at TE of 40 ms and 3 T (Provencher, 1993). Concentrations of NAA, Glx, tCr, tCho, and Myo were estimated in millimoles per liter (mmol/L) as means of the four individual spectra from the four contiguous individual voxels for each hippocampus, yielding a spectrum from a voxel size of 15 × 20 × 20 mm^3^ (Figure 2).

To account for partial volume effects of CSF, GM, and WM within the MRS voxels, a multi‐voxel segmentation method was employed so that fractional tissue quantities could be obtained to enable tissue‐fraction correction of metabolites concentration estimates (further described in Supporting Information 1) (Quadrelli et al., 2016). MRS quality control was ensured by excluding individual voxel spectra if water peak FWHM during shimming >25 Hz, Cramer‐Rao lower bounds (i.e., % standard deviations) >30%, or partial CSF fraction >40%. Spectral qualities of the VOI were validated in comparison with a previously published MRS reproducibility study (Wiebenga et al., 2014).

#### 2.4.2 Cardiorespiratory fitness

CRF was assessed using the 20 m multistage fitness test, which has good validity in adolescents and is the most widely accepted field‐based measure of CRF (Castro‐Piñero et al., 2010; Lang et al., 2018). Verbal encouragement was provided by assessors blinded to group allocation. The last successful stage was recorded and converted to the number of laps completed.

#### 2.4.3 Muscular fitness

The 90‐degree push‐up and standing long jump tests were used to assess upper body muscular endurance and lower body muscular power, respectively (Lubans et al., 2011; Ruiz et al., 2011).

#### 2.4.4 Body composition

Body weight and height were measured using a portable digital scale (A&D Medical UC‐352‐BLE Digital Scales) and stadiometer (Seca 213 Portable Height Measuring Rod Stadiometer), respectively. Body mass index (BMI) was calculated (weight[kg]/height[m]^2^) and the International Obesity Task Force cut‐offs were used to classify participants into weight categories (Cole & Lobstein, 2012).

#### 2.4.5 Physical activity

Participants were instructed to wear an ActiGraph GT9X Link accelerometer on their non‐dominant wrist for 24 h/day for seven consecutive days. Weekday moderate‐to‐vigorous physical activity (MVPA) and sedentary behavior (mean minutes/day) were calculated using existing cut‐points (Chandler et al., 2016).

#### 2.4.6 Working memory

Working memory was assessed using a serial 2‐back task completed in the classroom prior to fitness assessments on laptops installed with specialized software (PsychoPy) (Peirce et al., 2019; Shigeta et al., 2021). Stimuli (3 cm colored shapes) were presented focally on a black background for 250 ms followed by an inter‐stimulus interval of 2500 ms. Response time and accuracy were recorded, and the *d*‐prime was calculated as an additional measure of accuracy (Shigeta et al., 2021). Further detail regarding the 2‐back task is described in Supporting Information 2.

### 2.5 Statistical analyses

Our brain region of interest was specified prospectively (Leahy et al., 2019), and data were analyzed by researchers blinded to group allocation using SPSS (version 27). Intervention effects were analyzed using linear mixed models including fixed effects for group (B2L or control), time (treated as categorical with levels baseline and 6‐months), and the group‐by‐time interaction [i.e., intervention post‐test mean minus intervention baseline mean) minus (control post‐test mean minus control baseline mean)], with a random intercept to account for the clustered nature of the data (i.e., students clustered within classes). The models were run with and without adjustment for sex (male or female) and body composition (BMI Z‐score). The effect sizes of difference in change over time were calculated using Cohen's *d*, while correlation coefficients between change scores were calculated using Spearman's tests.

## 3 RESULTS

**FIGURE 3:**
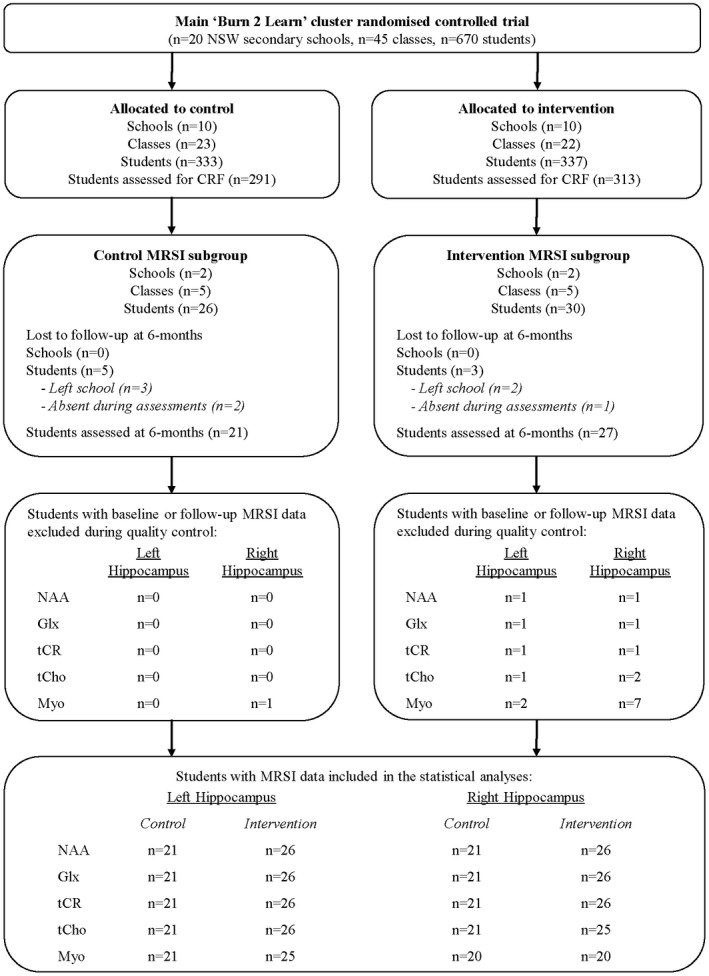
Flow of clusters, participants, and MRS data through the study

The flow of clusters, participants, and MRS data through the study is displayed in Figure 3. Of the 56 participants recruited, 48 (86%) participants were assessed at 6‐months follow‐up. Of the participants not followed, five (9%) permanently left their school and three (5%) were absent from school during assessments. The intervention group completed 2.9 ± 0.7 HIIT sessions each week at school.

### 3.1 Baseline characteristics

Participant demographic characteristics at baseline are detailed in Table 1. The intervention group consisted of 30 participants (73% female) with a mean age of 16.0 ± 0.2 years and the control group consisted of 26 participants (50% female) with a mean age of 16.1 ± 0.6 years. The two groups were balanced for all demographic characteristics at baseline.

**TABLE 1:** Participant demographic characteristics at baseline

Characteristic	Control (*n* = 26)	Intervention (*n* = 30)	Total (*n* = 56)
Age (years)	16.1 ± 0.6	16.0 ± 0.2	16.1 ± 0.4
Female	13 (50%)	22 (73%)	35 (61%)
Cultural background			
Australian	21 (81%)	27 (90%)	48 (86%)
European	1 (4%)	3 (10%)	4 (7%)
African	1 (4%)	–	1 (2%)
Asian	1 (4%)	–	1 (2%)
Middle Eastern	1 (4%)	–	1 (2%)
Other	1 (4%)	–	1 (2%)
Australian indigenous descent	3 (12%)	–	3 (5%)
Socioeconomic status			
Low/Medium	21 (81%)	21 (70%)	42 (75%)
High	5 (19%)	9 (30%)	14 (25%)
Weight status (BMI‐for‐age)			
Healthy weight	14 (54%)	21 (70%)	35 (63%)
Overweight	7 (27%)	5 (17%)	12 (21%)
Obese	5 (19%)	4 (13%)	9 (16%)
Physical activity			
Weekday MVPA (min)	42 ± 11	41 ± 17	42 ± 17
Weekday sedentary time (min)	476 ± 42	481 ± 61	479 ± 53
CRF (multistage fitness test laps)	32 ± 12	37 ± 14	34 ± 14
Upper body MF (push‐ups)	8 ± 7	8 ± 7	8 ± 7
Lower body MF (SLJ distance in cm)	164 ± 41	161 ± 31	162 ± 32

*Note*: Data are reported as mean ± standard deviation and counts (percentage).

Abbreviations: BMI, body mass index; cm, centimeters; CRF, cardiorespiratory fitness; MF, muscular fitness; min, minutes; MVPA, moderate‐vigorous physical activity; SLJ, standing long jump.

### 3.2 Changes in hippocampal metabolism

We observed group‐by‐time effects for NAA (+2.66 mmol/L, 95% confidence interval [CI] 0.20 to 5.11, *p* = .04, *d* = 0.66) and Glx (+3.38 mmol/L, 95% CI 0.34 to 6.42, *p* = .03, *d* = 0.67) concentrations in the left hippocampus after the B2L program compared to the control group (Figures S2 and S3). There were no other significant group‐by‐time effects for hippocampal metabolites. Within‐group effects for all metabolite concentrations were observed in the left hippocampus of the intervention group, consistent with our hypothesis. A summary of between‐ and within‐ group effects is presented in Table 2 (adjusted for sex and BMI Z‐score) and Table S1 (unadjusted). Adjusting for sex and BMI Z‐score in the models generally strengthened the magnitude of the effects observed in the unadjusted models.

**TABLE 2:** Changes in hippocampal metabolites at 6‐month follow‐up between participants randomized to wait‐list control or the B2L intervention adjusted for sex and weight‐status

Outcomes	No. of participants		Mean change from baseline (95% CI)		Adjusted difference at follow‐up^a^		
	Control	Intervention	Control	Intervention	Coefficient (95% CI)	Cohen's *d*	p
NAA (left)	21	26	1.09 (−0.85, 3.03)	3.81 (2.22, 5.41)**	2.66 (0.20, 5.11)*	0.66	.04
NAA (right)	21	26	0.59 (−0.57, 1.76)	1.86 (0.84, 2.87)**	1.26 (−0.28, 2.81)	0.49	.11
Glx (left)	21	26	−0.72 (−3.01, 1.58)	2.66 (0.68, 4.65)*	3.38 (0.34, 6.42)*	0.67	.03
Glx (right)	21	26	1.04 (−0.61, 2.68)	1.21 (−0.21, 2.62)	0.17 (−2.00, 2.34)	0.05	.88
tCr (left)	21	26	0.62 (−0.63, 1.88)	1.33 (0.24, 2.42)*	0.70 (−0.96, 2.37)	0.26	.40
tCr (right)	21	26	0.46 (−0.44, 1.36)	0.83 (0.05, 1.62)*	0.37 (−0.83, 1.56)	0.19	.54
tCho (left)	21	26	−1.66 (−2.01, −1.30)**	−1.45 (−1.75, −1.14)**	0.21 (−0.26, 0.68)	0.27	.37
tCho (right)	21	25	0.18 (−0.16, 0.51)	0.28 (−0.01, 0.58)	0.11 (−0.34, 0.56)	0.15	.63
Myo (left)	21	25	−0.42 (−3.16, 2.32)	−2.89 (−5.28, −0.50)*	−2.47 (−6.11, 1.18)	−0.41	.18
Myo (right)	20	20	2.06 (−10.22, 14.34)	−8.12 (−19.30, 3.06)	−10.18 (−26.76, 6.40)	−0.40	.22

Abbreviations: Glx, glutamate+glutamine; Myo, myo‐inositol; NAA, *N*‐acetylaspartate; tCho, total choline; tCr, total creatine.

^a^ Adjusted difference [(Intervention post‐test mean minus Intervention baseline mean) minus (Control post‐test mean minus Control baseline mean)] in metabolite concentrations (mmol/L).

* *p* < .05

** *p* < .01.

### 3.3 Correlations between changes in left hippocampal NAA and Glx concentrations with secondary outcomes

Changes in left NAA and Glx concentrations were not associated with any secondary outcomes in the whole sample. We examined if there were correlations between changes in left hippocampal NAA and Glx concentrations with secondary outcomes that were specific to the intervention (Table S2). In the B2L group, changes in CRF were positively associated with changes in left NAA (*r*_*s*_ = 0.52, *p* = .016) and Glx (*r*_*s*_ = 0.57, *p* = .007) concentrations (Figure S7). Changes in standing long jump distance were also positively associated with changes in left NAA (*r*_*s*_ = 0.47, *p* = .024) and Glx (*r*_*s*_ = 0.59, *p* = .003) concentrations. Additionally, changes in 2‐back target accuracy were positively associated with changes in left NAA (*r*_*s*_ = 0.41, *p* = .037) and Glx (*r*_*s*_ = 0.43, *p* = .028) concentrations (Figure S8). The increases in left hippocampal NAA and Glx concentrations were not associated with changes in any other secondary outcomes.

## 4 DISCUSSION

The aim of this study was to examine the effects of a HIIT intervention on hippocampal metabolism in a sample of lower fit older adolescents. The B2L program increased left hippocampal concentrations of NAA and Glx, and these changes correlated with changes in CRF, lower body muscular fitness, and working memory in the intervention group.

This study is the first to use MRS to elucidate the effects of physical activity on hippocampal metabolism in youth. Our findings are consistent with those observed in a three‐month exercise study in schizophrenia patients, which found an increase in hippocampal volume in the aerobic training group correlated with both improvements in CRF and increases in hippocampal NAA (Pajonk et al., 2010). A previous acute experimental study reported increased Glx concentration in the brains of healthy young adults after a bout of exercise at a similar intensity to the B2L intervention (~85% age‐predicted HR_max_) (Maddock et al., 2011), which replicates prior animal studies (van Praag, 2009). Maddock et al. suggest that non‐oxidized carbohydrate entering the brain during vigorous exercise may be partly directed toward increased synthesis of glutamatergic neurotransmitters, which could contribute to the therapeutic effects of exercise in people with mental health disorders. Speculatively, our findings suggest that repeated exposure to bouts of physical activity could lead to an accrual of Glx in the hippocampus over time, which may be able to offset/attenuate the decreased hippocampal Glx observed in these populations.

Our findings also add support for the benefits of physical activity on cognition (Biddle et al., 2019), as hippocampal levels of Glx are associated with memory (Nikolova et al., 2017), and typically decrease with aging, MCI, Alzheimer's disease, dementia with Lewy bodies, and schizophrenia (Huang et al., 2017; Rauchmann et al., 2020; Su et al., 2016). Cross‐sectional literature also links frontal cortex NAA and creatine concentrations with working memory in children and adolescents (Ozturk et al., 2009; Yeo et al., 2000). Frontal cortex NAA levels have also been shown to mediate the association between CRF and working memory in older adults (Erickson et al., 2012). Relatedly, frontal cortex NAA concentration was higher in endurance‐trained compared to sedentary adults, and was predicted by CRF (Gonzales et al., 2013). A recent cross‐sectional study in young‐middle aged adults also reports higher hippocampal NAA concentrations in those engaging in high (compared to low) levels of physical activity, along with associations between CRF and both hippocampal NAA concentration and volume (Hendrikse et al., 2022).

NAA is a neuronal marker which typically increases during development and decreases during aging (Hüppi et al., 1991; Rae, 2014; van der Knaap et al., 1990; Yang et al., 2015). Our findings indicate that increasing physical activity and fitness may lead to changes in cellular morphology (e.g., neurogenesis, dendritic branching, myelination) in the hippocampus above that typically observed during adolescence (Goddings et al., 2014). These findings align with earlier cross‐sectional associations of CRF with hippocampal volume and perfusion in youth, and experimental exercise findings in an animal model of adolescence (Chaddock et al., 2010; Chaddock‐Heyman et al., 2016; DiFeo & Shors, 2017; Esteban‐Cornejo et al., 2017; Herting & Nagel, 2012). Further, an acute experimental study found that moderate‐intensity aerobic exercise improved working memory and increased left hippocampus activation in children (Chen et al., 2016).

Therefore, although it remains to be established if physical activity can increase the volumetric growth of the hippocampus during childhood and adolescence in humans, multi‐modal MRI findings converge to support the impact of physical activity on hippocampal metabolism, perfusion, and activation. While many human studies have attempted to replicate animal models that have shown unequivocal links between physical activity, hippocampal neurogenesis, and brain‐derived neurotrophic factor (de Azevedo et al., 2020; Loprinzi, 2019), NAA and Glx may serve as more sensitive and stable biomarkers of in vivo changes in morphology in response to physical activity interventions.

### 4.1 Strengths and limitations

This is the first study to examine the impact of physical activity on hippocampal metabolism in older adolescents using MRS, and addresses the lack of adolescent samples within the experimental physical activity and neuroimaging literature (Valkenborghs et al., 2019). Additional study strengths include: (i) the evaluation of a physical activity intervention delivered in a real‐world setting with a high level of fidelity, (ii) prospectively defined a priori study hypotheses, and (iii) blinded outcome assessment and analysis procedures. The absolute quantification of metabolite concentrations, which has been shown to be superior to relative quantification in the hippocampus within the context of metabolites pertinent to cognitive health, is another study strength (Watanabe et al., 2008).

However, there are some limitations. First, it is possible that slight differences in voxel positioning at baseline and follow‐up could have led to partial volume contamination and influenced the metabolite concentrations observed. Second, because this study occurred in schools, we used field‐based measures of CRF and muscular fitness, as opposed to gold standard, laboratory‐based assessments (i.e., peak oxygen consumption and 1‐repetition maximum strength tests). Third, due to time constraints, we were unable to scan the prefrontal cortex as stipulated in our prospective trial registration. Finally, our sample size was relatively small and homogeneous, and schools (rather than individuals) were the unit of randomization. Nevertheless, our analyses were adjusted for clustering at the class level using a random intercept in our mixed models (school level clustering is negligible after adjustment for class level clustering) (Heo & Leon, 2008).

## 5 CONCLUSION

Our findings suggest that HIIT improves hippocampal metabolism in lower fit older adolescents. Specifically, we observed increased concentrations of NAA and Glx in the left hippocampus following a 6‐month school‐based physical activity intervention. Increases in left hippocampal NAA and Glx concentrations were associated with improvements in CRF, lower body muscular fitness, and working memory in the intervention group, which may indicate a potential neurobiological mechanism for the effect of physical activity on cognition. Future studies should employ larger sample sizes to replicate these findings and to investigate whether (a) changes in CRF and muscular fitness mediate changes in hippocampal metabolite concentrations, and (b) changes in hippocampal metabolite concentrations mediate the benefits of physical activity on working memory.

## AUTHOR CONTRIBUTIONS

**Sarah Ruth Valkenborghs:** Data curation; formal analysis; investigation; methodology; writing – original draft; writing – review and editing. **Charles H. Hillman:** Funding acquisition; methodology; resources; software; writing – review and editing. **Oun Al‐Iedani:** Data curation; formal analysis; investigation; methodology; visualization; writing – review and editing. **Michael Nilsson:** Funding acquisition; methodology; writing – review and editing. **Jordan J. Smith:** Funding acquisition; investigation; methodology; resources; writing – review and editing. **Angus Aaron Leahy:** Data curation; investigation; resources; writing – review and editing. **Simon K. Harries:** Data curation; writing – review and editing. **Saadallah Ramadan:** Methodology; resources; software; writing – review and editing. **David Revalds Lubans:** Conceptualization; funding acquisition; investigation; methodology; resources; supervision; writing – original draft.

## CONFLICT OF INTEREST

The authors have no conflicts of interest relevant to this article to disclose.

## Data Availability

Requests for access to de‐identified data from the study should be addressed to the corresponding author (david.lubans@newcastle.edu.au). All proposals requesting data access will need to specify how it plans to use the data, and all proposals will need approval of the trial co‐investigator team before data release.
