# Supplementary material for: Effect of high‐intensity interval training on hippocampal metabolism in older adolescents
Source: Psychophysiology. 2022 May 22;59(11):e14090. doi: 10.1111/psyp.14090 (PMC9787522; doi:10.1111/psyp.14090)
Supplement: Supplementary file 1 [file PSYP-59-e14090-s001.docx]

## Supplemental Materials

**Supplemental Information 1 – Individual MRS voxel segmentation**

To account for the partial volume effects of CSF, GM, and WM within the MRSI voxels, a multi-voxel segmentation method was applied. The centroid coordinates of each MRS voxel were determined from the MRS VOI centroid coordinates reported in the Siemens exported ‘.rda’ file (VOIPositionSag, VOIPositionCor, VOIPositionTra and VOIPhaseFOV, VOIReadoutFOV, VOIThickness). An ‘.rda’ file was generated for each MRS voxel based on its computed centroid, as calculated using an in-house MATLAB code (2018b, The MathWorks, Inc., Natick, MA, USA).

The “mask” function within the SPM toolbox was employed to create binary masks of the MRS VOI (80x80 mm^2^) and each 1x1x1.5 cm^3^ MRS voxel (Supplemental Figure 1). FSL FAST was used to create partial volume masks for the MRS VOI and each MRS voxel so that fractional quantities of CSF, GM, and WM could be obtained. The existing in-house MATLAB code was modified for multi-voxel ‘.rda’ file inputs so that the MRS voxels could be co-registered with the T1-MPRAGE. The original MRS VOI could then be re-constructed by overlaying all of the MRS voxels on the T1-MPRAGE simultaneously. Summation of the total fractional quantities from the multi-voxels (2 × 2) and comparison to the total fractional quantities of the VOI was also used to validate this multi-voxel segmentation approach.

**Supplemental Information 2 – 2-back task**

Working memory was assessed using a serial 2-back task to evaluate differences in the degree of cognitive demand. In this task, participants were presented with a series of six basic shapes (i.e., square, star, circle, cross, crescent, triangle) and required to recall (using the ‘Q’ key with the left index finger and the ‘P’ key with the right index finger) whether the shape currently displayed (trial ‘n’) matched the shape two shapes prior (2-back, trial ‘n-2’). For each trial, participants were required to indicate whether the shape was a match (i.e., target) or not a match (i.e., non-target). Shapes were presented for a duration of 250 ms following a fixed interstimulus interval of 2500 ms. Following task instructions, participants completed a practice block consisting of 20 trials to ensure understanding. If overall accuracy was below 70%, participants completed another practice block, and the test administrator confirmed their understanding of the test. The task consisted of two blocks of randomised target (n=24) and non-target (n=48) trials which were presented in a counterbalanced order and had an approximate run time of 10 minutes. Response time and accuracy were recorded for both target shapes (i.e., correctly identifying a match) and non-target shapes (i.e., correctly identifying a non-match). Additionally, the relative proportion of ‘hits’ (correct target trials) to ‘false alarms’ (incorrect selection on target trial), otherwise known as the d-prime score, was calculated for target and non-target trials. A higher d-prime score reflects a greater capacity to differentiate target from non-target shapes when performing the n-back tasks, thereby indicating greater working memory.

**Supplemental Figures**

Supplemental Figure 1. Pipeline of volumetric brain tissue segmentation from MRI and MRS data. A) A binary mask of a MRS single slice VOI (8x8x1.5cm^3^) was created using the SPM toolbox. B) partial volume masks for each tissue type were created using FSL FAST. C) Tissue segmentation of MRS VOI (CSF, GM and WM) and hippocampal voxels overlaid on the T1 image.


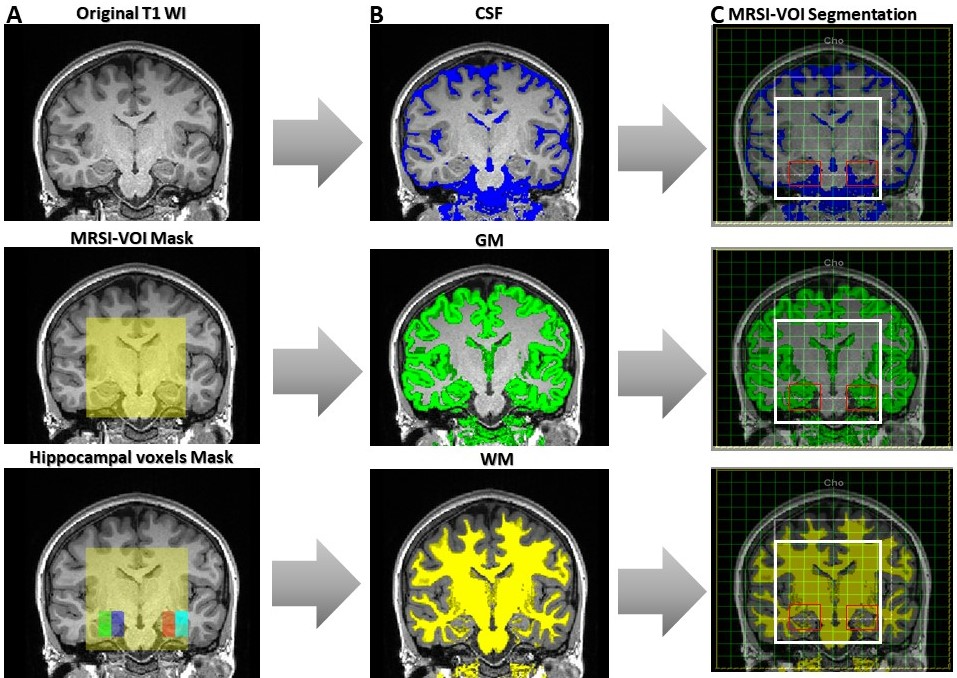


Supplemental Figure 2. Error bar chart of pre-post changes in NAA concentrations in the left and right hippocampus per group.


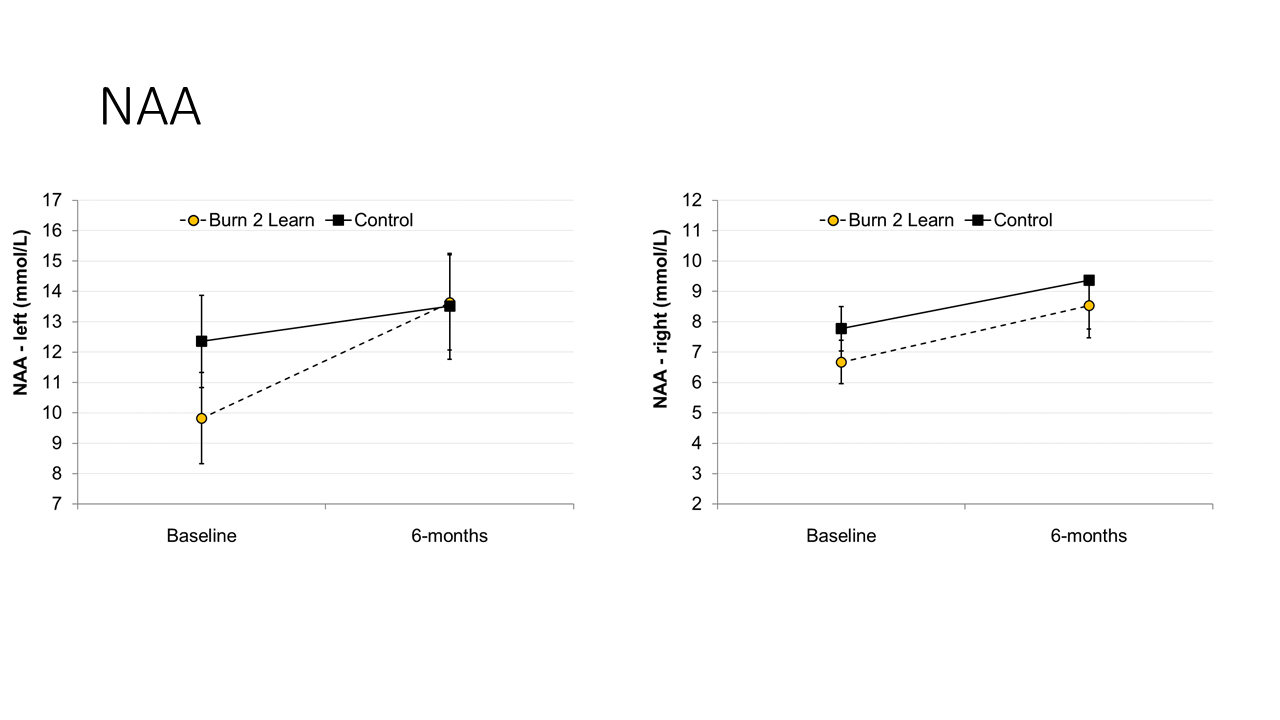


Supplemental Figure 3. Error bar chart of pre-post changes in Glx concentrations in the left and right hippocampus per group.


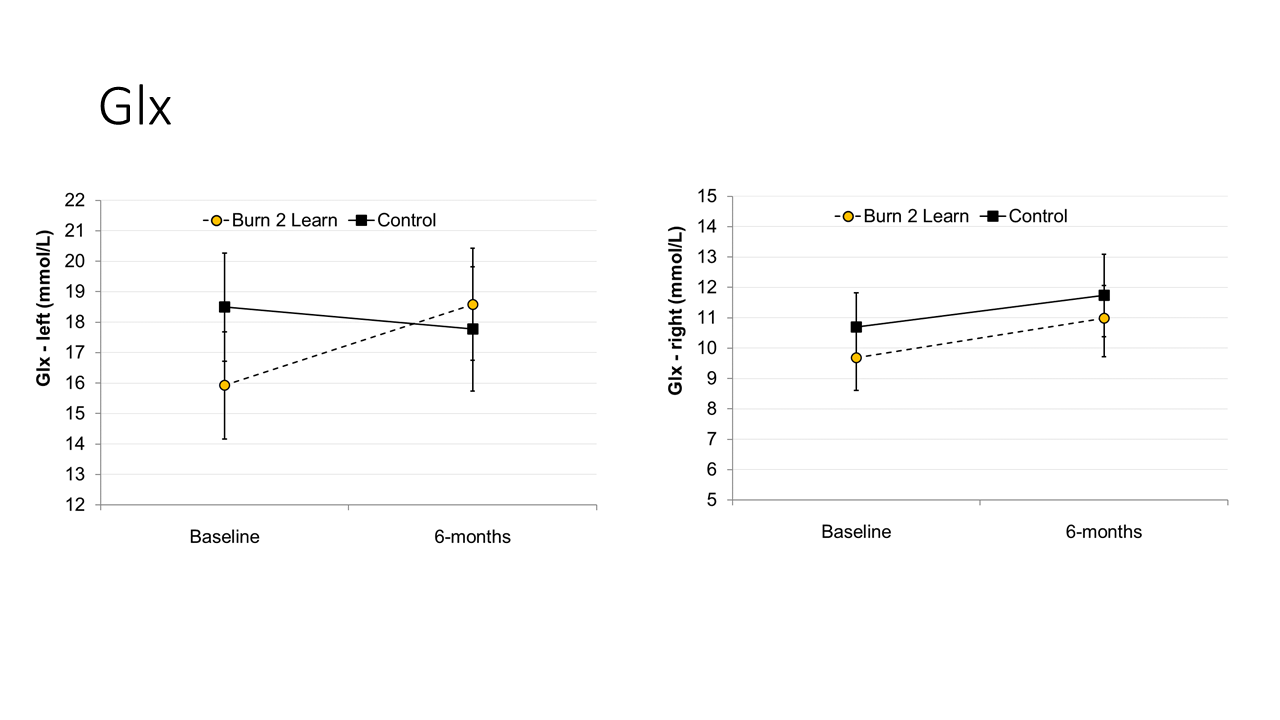


Supplemental Figure 4. Error bar chart of pre-post changes in Myo concentrations in the left and right hippocampus per group.


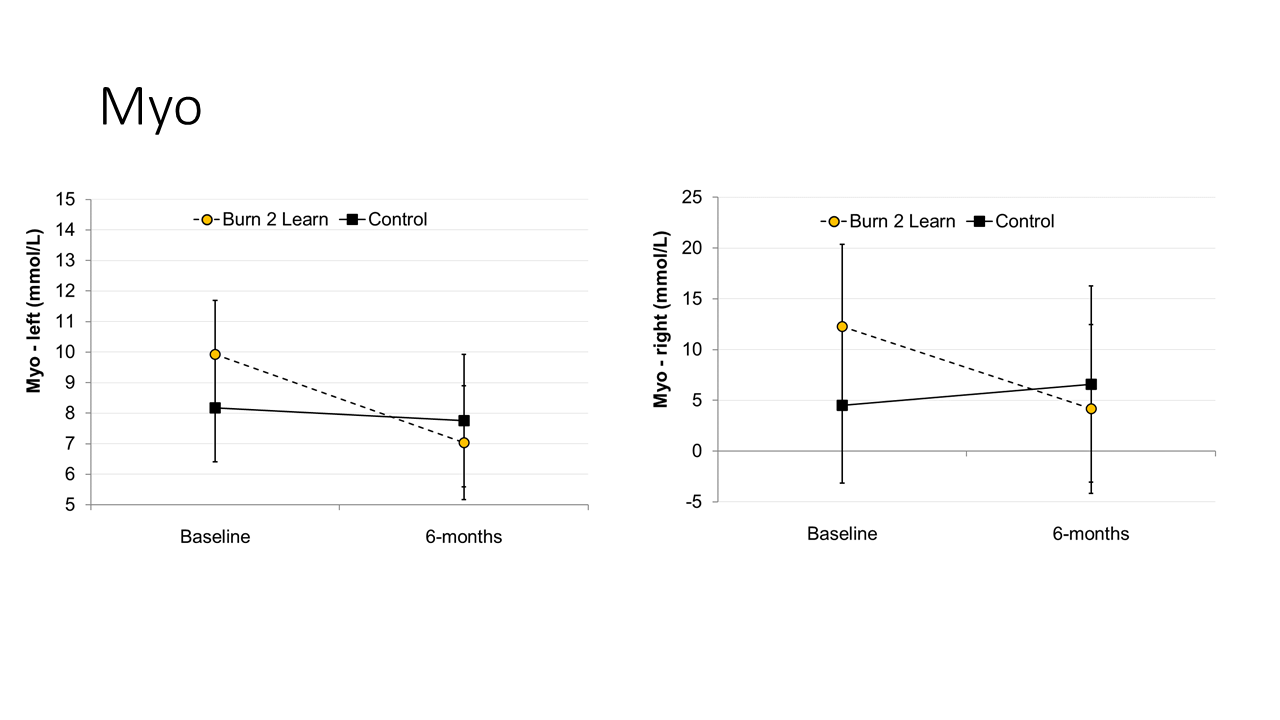


Supplemental Figure 5. Error bar chart of pre-post changes in tCho concentrations in the left and right hippocampus per group.
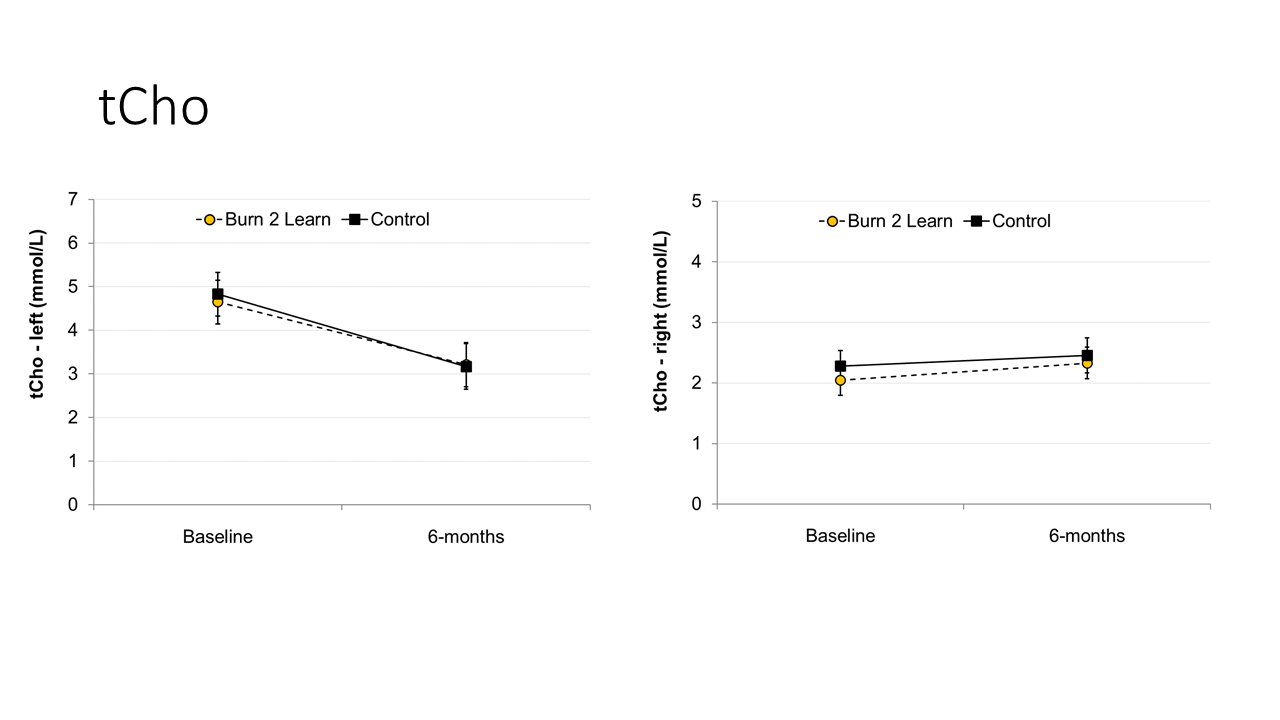


Supplemental Figure 6. Error bar chart of pre-post changes in tCr concentrations in the left and right hippocampus per group.


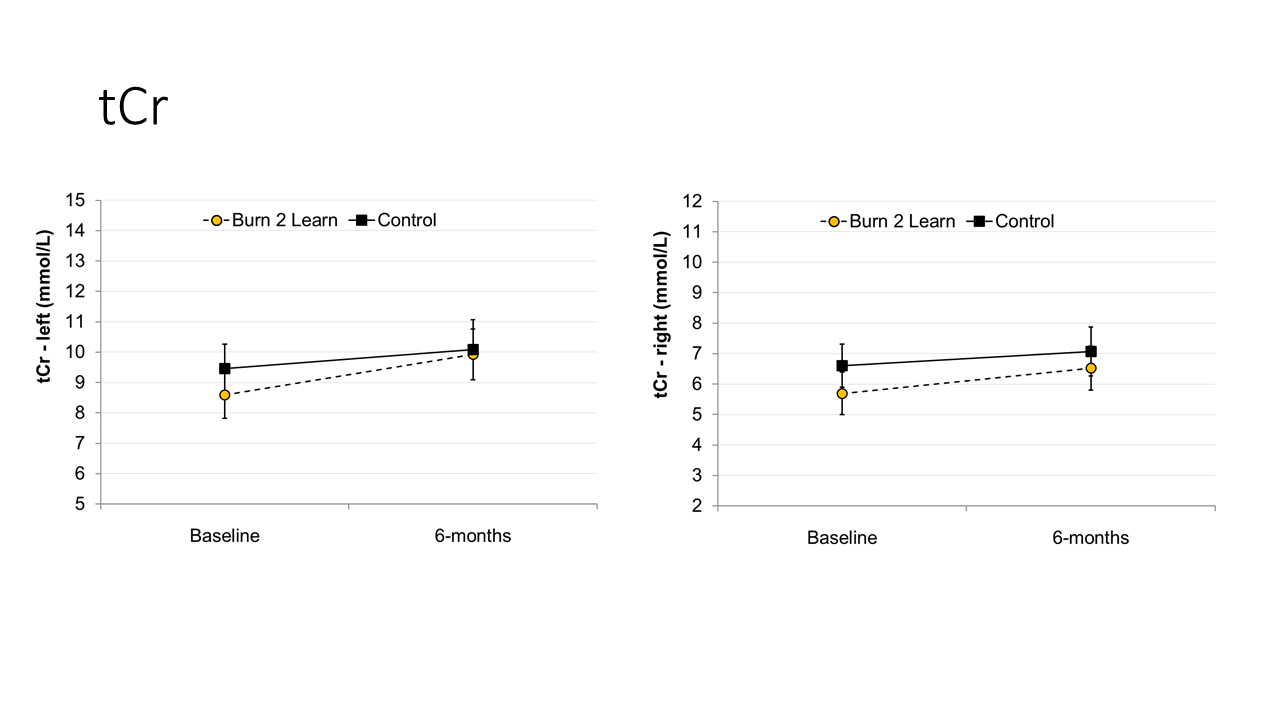


Supplemental Figure 7. Scatterplot of associations between changes in cardiorespiratory fitness with changes in left hippocampal concentrations of (A) NAA and (B) Glx in the intervention group.


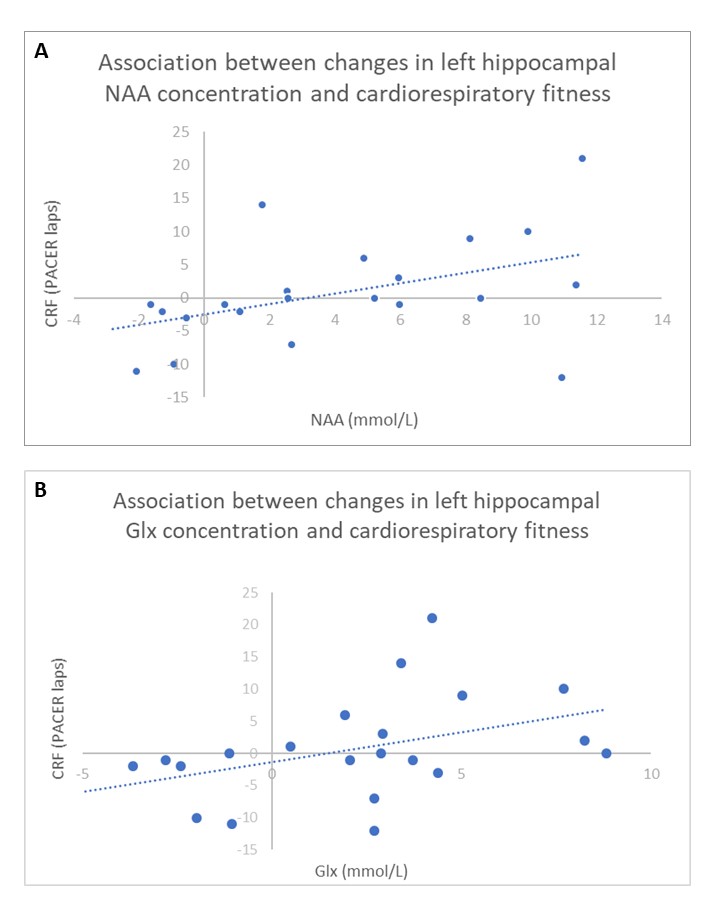


Supplemental Figure 8. Scatterplot of associations between in changes in 2-back target accuracy with changes left hippocampal concentrations of (A) NAA, (B) Glx and (C) tCr in the intervention group.
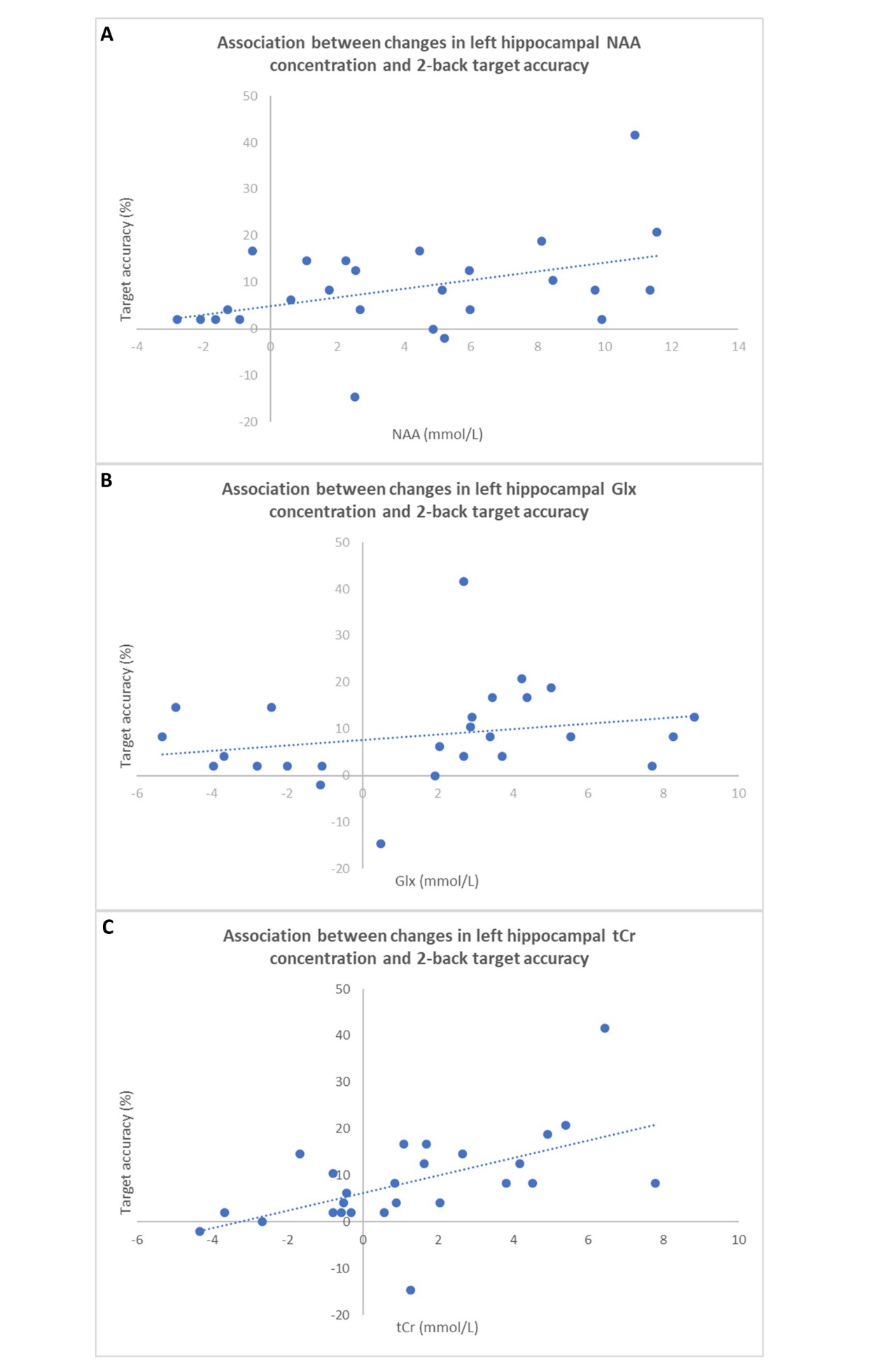


Supplemental Table 1. Changes in hippocampal metabolites at 6-month follow-up between participants randomized to wait-list control or the B2L intervention (without covariates)

| **Outcomes** | **No. of participants** | | **Mean change from baseline (95% CI)** | | **Adjusted difference at follow-up^1^** | | |
| --- | --- | --- | --- | --- | --- | --- | --- |
|  | **Control** | **Intervention** | **Control** | **Intervention** | **Coefficient (95% CI)** | **Cohen’s *d*** | **p** |
| NAA (left) | 21 | 26 | 1.62 (-0.06, 3.30) | 3.85 (2.33, 5.38)** | 2.23 (-0.04, 4.50) | 0.60 | 0.05 |
| NAA (right) | 21 | 26 | 0.81 (-0.26, 1.89) | 1.86 (0.88, 2.84)** | 1.04 (-0.41, 2.50) | 0.43 | 0.16 |
| Glx (left) | 21 | 26 | -0.15 (-2.25, 1.96) | 2.67 (0.75, 4.58)** | 2.82 (-0.03, 5.66) | 0.60 | 0.05 |
| Glx (right) | 21 | 26 | 1.15 (-0.38, 2.67) | 1.22 (-0.18, 2.61) | 0.07 (-1.99, 2.13) | 0.02 | 0.95 |
| tCr (left) | 21 | 26 | 8.50 (7.76, 9.24) | 9.81 (9.01, 10.60)* | 0.49 (-1.08, 2.06) | 0.19 | 0.53 |
| tCr (right) | 21 | 26 | 0.47 (-0.38, 1.32) | 0.83 (0.06, 1.61)* | 0.36 (-0.79, 1.51) | 0.19 | 0.53 |
| tCho (left) | 21 | 26 | -1.63 (-1.96, -1.31)** | -1.44 (-1.73, -1.15)** | 0.19 (-0.24, 0.63) | 0.27 | 0.37 |
| tCho (right) | 21 | 25 | 0.16 (-0.16, 0.48) | 0.29 (-0.00, 0.58) | 0.13 (-0.30, 0.56) | 0.19 | 0.54 |
| Myo (left) | 21 | 25 | -0.33 (-2.86, 2.21) | -2.93 (-5.26, -0.60) | -2.60 (-6.04, 0.84) | -0.46 | 0.14 |
| Myo (right) | 20 | 20 | 1.37 (-10.00, 12.74) | -8.10 (-19.02, 2.82) | -9.47 (-25.24, 6.30) | -0.39 | 0.23 |

Glx = glutamate+glutamine; Myo = myo-inositol; NAA = *N*-acetylaspartate; tCho = total choline; tCr = total creatine.

^1^Adjusted difference [(Intervention post-test mean minus Intervention baseline mean) minus (Control post-test mean minus Control baseline mean)] in metabolite concentrations (mmol/L).

***p* < 0.01; **p* < 0.05.

Supplemental Table 2. Changes in secondary outcomes at 6-month follow-up between participants randomized to wait-list control or the B2L intervention adjusted for sex and weight-status

| **Secondary outcomes** | **No. of participants** | | **Mean change from baseline (95% CI)** | | **Adjusted difference at follow-up^1^** | | |
| --- | --- | --- | --- | --- | --- | --- | --- |
|  | **Control** | **Intervention** | **Control** | **Intervention** | **Coefficient (95% CI)** | **Cohen’s *d*** | **p** |
| Cardiorespiratory fitness (laps) | 21 | 22 | -2.08 (-6.51, 2.35) | 0.24 (-3.54, 4.02) | 2.32 (-3.48, 8.12) | 0.25 | 0.42 |
| Upper body muscular endurance (reps) | 17 | 25 | -1.21 (-3.50, 1.08) | -0.38 (-2.14, 1.39) | 0.84 (-2.04, 3.71) | 0.19 | 0.56 |
| Lower body muscular power (cm) | 19 | 24 | -4.67 (-13.78, 4.44) | -8.96 (-16.90, -1.02)* | -4.29 (-16.32, 7.73) | -0.23 | 0.47 |
| BMI (Z-score) | 21 | 28 | -0.31 (0.17, 0.11) | 0.16 (0.04, 0.29)* | 0.19 (0.01, 0.38)* | 0.61 | 0.04 |
| MVPA (min/weekday) | 13 | 20 | -14.23 (-20.46, -8.01)** | -5.20 (-11.51, 1.11) | 9.04 (0.20, 17.87)* | 0.75 | 0.05 |
| Sedentary time (min/weekday) | 13 | 20 | 10.11 (-13.66, 33.88) | 22.52 (-1.527, 46.56) | 12.41 (-21.27, 56.09) | 0.27 | 0.46 |
| 2-back target accuracy (%) | 21 | 28 | 12.51 (5.03, 19.98)** | 8.51 (2.02, 15.01)* | -4.00 (-13.88, 5.89) | -0.24 | 0.42 |
| 2-back non-target accuracy (%) | 21 | 28 | 14.84 (7.39, 22.28)** | 5.64 (-0.90, 12.17) | -9.20 (-19.10, 0.69) | -0.54 | 0.07 |
| 2-back target reaction time (ms) | 21 | 28 | -55.95 (-134.22, 22.38) | -83.90 (-151.24, -16.54)* | -27.95 (-130.95, 75.06) | -0.16 | 0.59 |
| 2-back non-target reaction time (ms) | 21 | 28 | -56.91 (-150.20, 36.38) | -95.37 (-175.44, -15.29)* | -38.46 (-161.06, 84.15) | -0.18 | 0.53 |
| 2-back *d*-prime | 21 | 28 | 0.22 (-0.24, 0.67) | 0.05 (-0.35, 0.45) | -0.17 (-0.77, 0.44) | -0.16 | 0.58 |

cm = centimeter; min = minute; ms = millisecond; reps = repetitions; % = percentage;

^1^Adjusted difference [(Intervention post-test mean minus Intervention baseline mean) minus (Control post-test mean minus Control baseline mean)] in metabolite concentrations (mmol/L).

***p* < 0.01; **p* < 0.05.

Supplemental Table 3. Correlations between changes in hippocampal metabolism and secondary outcomes in the intervention group

|  | CRF | Upper  body MF | Lower  body MF | BMI  Z-score | MVPA | Sedentary time | 2-back target acc | 2-back non-target acc | 2-back target RT | 2-back non-target RT | 2-back *d*-prime |
| --- | --- | --- | --- | --- | --- | --- | --- | --- | --- | --- | --- |
| NAA (left) | **0.52*** | -0.17 | 0.47 | 0.08 | -0.15 | -0.07 | **0.42*** | 0.29 | 0.28 | 0.06 | 0.36 |
| NAA (right) | 0.20 | -0.32 | 0.03 | 0.01 | -0.17 | -0.01 | 0.09 | 0.05 | 0.05 | 0.17 | -0.04 |
| Glx (left) | **0.57**** | -0.16 | 0.59 | 0.05 | 0.03 | -0.17 | **0.43*** | 0.30 | 0.27 | -0.01 | 0.35 |
| Glx (right) | 0.12 | 0.14 | 0.30 | 0.00 | 0.21 | -0.29 | 0.25 | 0.25 | 0.09 | -0.16 | 0.26 |
| tCr (left) | 0.22 | 0.13 | 0.36 | 0.10 | -0.26 | -0.01 | **0.64**** | 0.25 | 0.04 | -0.28 | 0.38 |
| tCr (right) | 0.31 | -0.31 | 0.20 | 0.07 | 0.09 | -0.20 | 0.14 | 0.13 | 0.13 | -0.03 | 0.10 |
| tCho (left) | -0.01 | -0.10 | 0.08 | -0.32 | 0.14 | -0.40 | 0.00 | 0.20 | -0.20 | -0.20 | 0.10 |
| tCho (right) | -0.05 | -0.12 | -0.06 | 0.20 | -0.22 | 0.05 | 0.17 | -0.03 | 0.06 | -0.04 | -0.04 |
| Myo (left) | -0.39 | 0.12 | 0.06 | -0.31 | 0.22 | -0.06 | -0.21 | -0.30 | 0.02 | -0.15 | -0.20 |
| Myo (right) | -0.22 | 0.21 | 0.18 | -0.40 | 0.39 | -0.43 | -0.03 | 0.34 | -0.25 | -0.43 | 0.31 |

Acc = accuracy; BMI = body mass index; CRF = cardiorespiratory fitness; Glx = glutamate+glutamine; MF = muscular fitness; MVPA = moderate-vigorous physical activity; Myo = myo-inositol; NAA = *N*-acetylaspartate; RT = reaction time; tCho = total choline; tCr = total creatine.

***p* < 0.01; **p* < 0.05.

Supplemental Table 4. Correlations between changes in hippocampal metabolites in the intervention group

| **Left** |  |  |  |  |  | **Right** |  |  |  |  |  |
| --- | --- | --- | --- | --- | --- | --- | --- | --- | --- | --- | --- |
|  | NAA | Glx | tCr | tCho | Myo |  | NAA | Glx | tCr | tCho | Myo |
| NAA |  |  |  |  |  |  |  |  |  |  |  |
| Glx | **0.74**** |  |  |  |  |  | 0.24 |  |  |  |  |
| tCr | **0.46*** | **0.68**** |  |  |  |  | **0.67**** | **0.65**** |  |  |  |
| tCho | 0.03 | 0.20 | 0.31 |  |  |  | **0.49*** | 0.32 | **0.69**** |  |  |
| Myo | **-0.46*** | -0.12 | -0.03 | 0.28 |  |  | **-0.57**** | 0.07 | -0.21 | **-0.46*** |  |

Glx = glutamate+glutamine; Myo = myo-inositol; NAA = *N*-acetylaspartate; tCho = total choline; tCr = total creatine.

***p* < 0.01; **p* < 0.05.
